# Development of experimental isolated lung perfusion model using surgically resected tumorous human lobes

**DOI:** 10.1186/s12890-026-04512-8

**Published:** 2026-07-21

**Authors:** Áron Bertram Gellért, Katalin Dezső, Melinda Rezeli, Zsolt Horváth, Sándor Paku, Bence Ferencz, Gyenge Bernát, Kristóf Csende, Hanna Tihanyi, Gábor Tarsoly, Márton Csaba, Sára Lality, Balázs Gieszer, Levente Bogyó, Klára Török, Péter Radeczky, Sára Radványi, Ákos Kocsis, László Agócs, Krisztina Bogos, Lilla Horváth, János Fillinger, Szilvia Török, Anna Sólyom-Tisza, Helga Kiss, András Czirók, Jenő Elek, Ildikó Madurka, Ferenc Rényi-Vámos, Balázs Döme, Zsolt Megyesfalvi, Áron Ghimessy

**Affiliations:** 1 Department of Thoracic Surgery, Semmelweis University and National Institute of Oncology, Ráth György utca. 7-9., Budapest, 1122 Hungary; 2 Department of Pulmonology, Department of Thoracic Surgery, Department of Pathology, Department of Anesthesiology and Intensive Therapy, Department of Tumor Biology, National Koranyi Institute of Pulmonology, Budapest, Hungary; 3 Department of Pathology and Experimental Cancer Research, Semmelweis University, Üllői út 26, Budapest, 1085 Hungary; 4 Department of Anesthesiology and Intensive Therapy, National Institute of Oncology, Budapest, Hungary; 5 Department of Ophthalmology, St. John Hospital, Budapest, Hungary; 6 Department of Biomedical Engineering, Lund University, Lund, Sweden; 7 Division of Thoracic Surgery, Department of Surgery, Medical University Vienna, Vienna, Austria; 8 Department of Biological Physics, Eötvös University, Budapest, Hungary; 9 Department of Translational Medicine, Lund University, Lund, Sweden

**Keywords:** Lung, Cancer, Human, Ex vivo perfusion

## Abstract

**Background:**

Ex vivo lung perfusion (EVLP) is a unique platform for lung assessment and preservation. While EVLP has been extensively investigated in donor lungs, its application to translational research is less well-defined. We aimed to evaluate and characterise the EVLP methodology using resected human cancerous lung.

**Methods:**

Between January 2021 and March 2023, 216 patients at our Institute underwent eligibility screening for experimental inclusion. Following pneumonectomy or lobectomy, 10 cases were included. The lung specimens were inflated, flushed with Perfadex^®^, and connected to an acellular perfusate circuit primed with Steen^®^ solution. Physiological parameters were monitored at regular intervals with a blood gas analyser.

**Results:**

The median duration of the experiments performed was 159 min, during which the functional parameters remained stable despite the progressive development of oedema. Importantly, elevated lactate levels did not correlate with functional deterioration defined by partial O_2_ and CO_2_ pressure levels despite a temporal non-significant decline in these parameters. We managed to perfuse pneumonectomy and lobectomy specimens with comparable results regarding the investigated physiological parameters. However, pneumonectomies were associated with shorter median warm ischemic times compared to lobectomies (29.5 min vs. 75 min, respectively).

**Conclusion:**

In this study, we have characterised proof-of-concept EVLP in cancerous human lungs regarding physiological parameters. Oedema management and clearly defined endpoints are essential in its translational application. These preliminary observations are intended to guide future investigators for improved protocols and establish standardised criteria for patient selection.

## Background

Ex Vivo lung perfusion (EVLP) is a rapidly evolving technique used as both an organ assessment tool for lung transplantation and a translational research technique to study the lung [1, 2]. In a clinical setting, distinct protocols have been developed in parallel, and the use of EVLP has successfully expanded the pool of donors for lung transplantation. In this context, EVLP serves to optimise transplant logistics by saving marginal donors [2]. In the most recent advancements, researchers use the platform to further develop existing strategies for transplantational EVLPs [3–5]. Novel, proof-of-concept therapeutic approaches regarding treatment and diagnostic of thoracic malignancies and inflammatory lung diseases are being validated on EVLP platforms [6–8]. During EVLP the lung is kept in nearly physiological conditions, which renders it an unparalleled method to study living human tissue. Notably, this is the only platform where human tumorous lung can be studied under near physiological conditions, without consequences on the human itself. It can be employed in future pharmaceutical research and there are notable attempts to do so [7, 8]. The development of in vivo lung perfusion techniques is as longstanding as that of EVLP, though their clinical effectiveness has yet to be fully demonstrated in lung cancer treatment [9, 10]. 

However, numerous challenges must be addressed to achieve optimal results. The experimental setting is highly equipment- and resource-intensive. This approach is most effective in high-volume thoracic surgery centres, where surgeons have extensive experience with lung transplantation, and a sufficient number of cases are available for review. In earlier experiments, histological examinations indicated that EVLP did not alter histopathological grading or staging, although this topic was first discussed nearly three decades ago [11]. Since then, pathological and genomic profiling have become routine in lung cancer phenotyping and genotyping as new therapies increasingly rely on these analyses [12]. Consequently, this aspect now needs to be reassessed.

Since there are currently no established guidelines defining the ideal research specimens or the specific physiological parameters to target in these experiments, this study aimed to establish a novel EVLP program focusing on tumorous lung tissue in order to gain hands-on experience and explore the potential applications and limitations of the experimental environment. In the present work, we focus exclusively on the physiological results from our cohort.

## Methods

### Patient selection

Between January 1st 2021 and March 31st 2023, we screened 216 cases who were scheduled for pneumonectomy or lower lobectomy at the National Korányi Institute of Pulmonology, Budapest, Hungary. We included cases where positron emission tomography-computed tomography (PET-CT) scans revealed fluorodeoxyglucose (FDG) avid nodules within the lung parenchyma and where attending physicians held a strong suspicion of lung cancer based on morphology and the patient’s clinical presentation. Cases in which the presence of a tumour caused atelectasis or circulatory disturbance, as determined by computed tomography (CT) scans, were excluded from the study. Patients with a history of thoracic irradiation or neoadjuvant chemotherapy were additionally excluded from the study. Out of those cases, 23 patients were selected and provided informed consent to participate in this study and subsequently underwent pneumonectomy or lobectomy. Following surgery, more than half of the cases (56.5%) were deemed unsuitable for the experiment due to visible tears or damage incurred during extraction. Other reasons for exclusion included anatomical variation and insufficiently long arterial or venous stumps to permit adequate anastomosis. From these, we successfully included 10 cases in our EVLP program (43.5%). There were seven male and three female participants. The patient cohort included six pneumonectomies and four lobectomies. Experiments’ characteristics can be seen in Table 1. In three cases, histological verification was not performed prior to surgery. The study was approved by the national ethics committee (Hungarian Scientific and Research Ethics Committee of the Medical Research Council (*ETT TUKEB IV/8075-3/2020/EKU*).

**Table 1:** Experiments’ characteristics

	Patient #1	Patient #2	Patient #3	Patient #4	Patient #5	Patient #6	Patient #7	Patient #8	Patient #9	Patient #10
Operation type	right side pneumonectomy	left side pneumonectomy	left side pneumonectomy	right side pneumonectomy	left side pneumonectomy	left lower lobectomy	left side pneumonectomy	left lower lobectomy	right lower lobectomy	right lower lobectomy
Lung cancer type	squamous cell carcinoma	squamous cell carcinoma	squamous cell carcinoma	squamous cell carcinoma	squamous cell carcinoma	lung adenocarcinoma	squamous cell carcinoma	lung adenocarcinoma	lung adenocarcinoma	small cell lung cancer
Stage (TMN 8th edition)	IIIA	IIA	IIIA	IIIA	IIB	IB	IIIA	IIA	IIIA	IIB
TNM descriptors	pT3 N1 M0	pT2b N0 M0	pT1c N2 M0	pT2a N2 M0	pT2a N1 M0	pT2a N0 M0	pT3 N1 M0	pT2b N0 M0	pT2a N2 M0	pT3 N0 M0
Age [years]	50–59	70–79	60–69	60–69	60–69	60–69	70–79	60–69	50–59	60–69
Height [meters]	1.8	1.76	1.72	1.97	1.78	1.7	1.77	1.48	1.55	1.72
Warm ischemic time [minutes]	10	34	10	40	25	20	45	75	86	46
Cold ischemic time [minutes]	95	99	75	45	75	49	28	50	49	70
Total ischemic time [minutes]	105	133	85	85	100	69	73	125	135	116
Experiment time [minutes]	158	228	160	222	133	162	137	150	179	140

### Specimen preparation

**Fig. 1:**
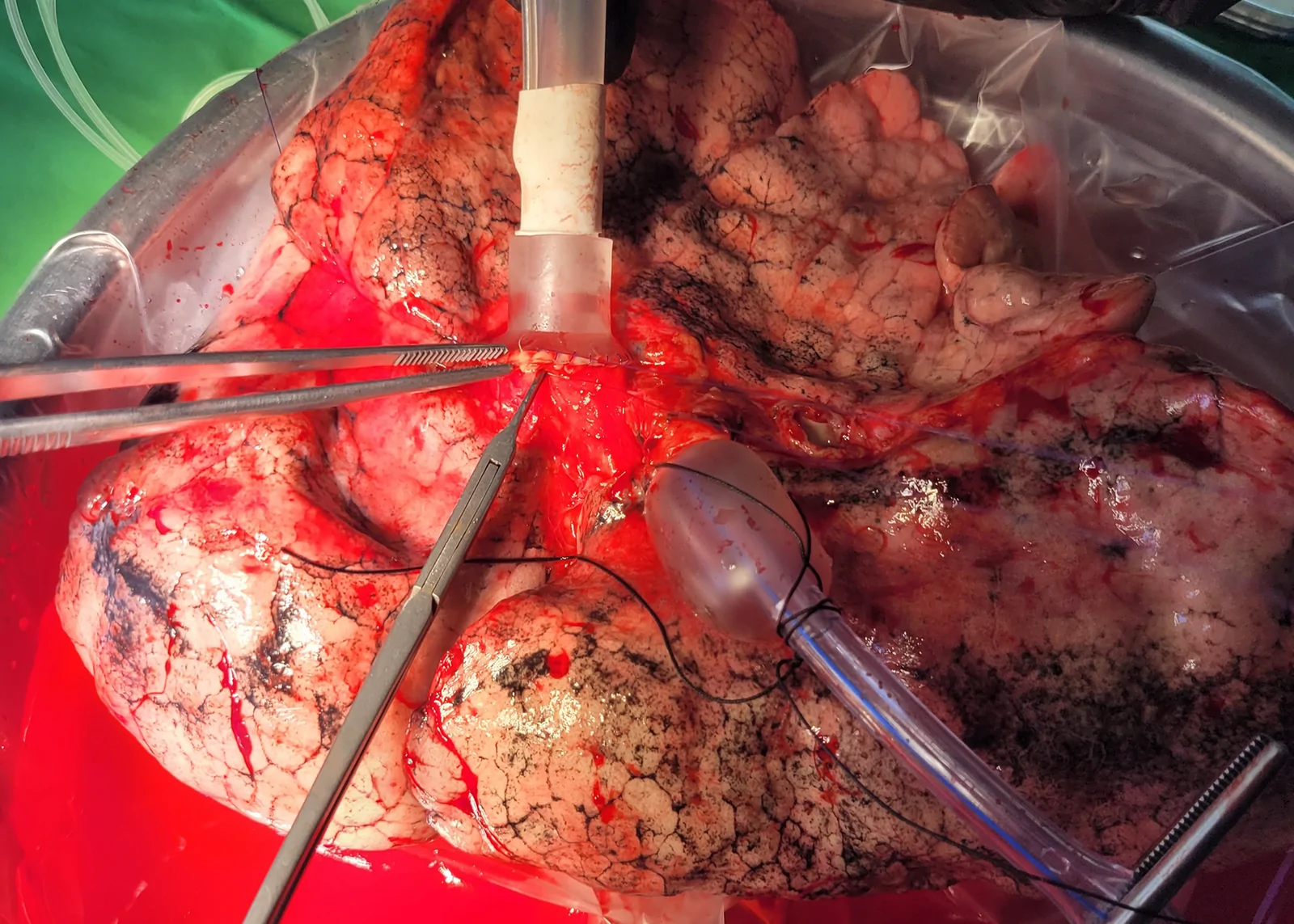
Close-up view of the cannula being sutured to the lung’s lobar artery

Surgery was conducted in accordance with established standard thoracic surgical procedures: either via open thoracotomy or video-assisted thoracoscopic surgery (VATS). After retrieval, the pulmonary lobe or lung was submerged in 4 °C saline solution, similar to the protocol used for donor organ preservation in lung transplantation procedures [13]. Bronchi and vessels were deliberately opened if they had been sealed with a stapler device; the bronchial and vascular resection margins were submitted for pathological examination. A veterinary-sized tracheal tube was inserted into the main bronchi and was fixed with a ligature knotted outside of the airway. The specimen was inflated using a silicone resuscitator ball, and the tube was clamped to keep approximately 40–50 cmH_2_O positive air pressure inside. This method helps preserve the lung by keeping alveoli open. Lung inflation is a critical step in the preservation and transportation of donor lungs [13, 14]. If the parenchyma sustained damage leading to an inability to maintain positive pressure, or vessels were deemed unsuitable for anastomosis, the organ was declared unfit for inclusion. In its inflated and cooled state (4 °C), the vasculature was flushed both retrograde and anterograde with 500 ml of 4 °C Perfadex^®^ solution (Xvivo Perfusion, Mölndal, Västra Götaland County, Sweden) to evacuate blood and possible thrombi. The lobar artery -and, if necessary, S6 segmental artery via a Y anastomosis- was sutured to the XVIVO Lung Cannula Set^®^ (Xvivo Perfusion, Mölndal, Västra Götaland County, Sweden) as shown in Fig. 1. Inserting cannulas into the pulmonary vein to create a closed circuit resulted in greater oedema formation in the specimen. Additionally, closing the pulmonary veins with a stapling device during surgery significantly increased the risk of subsequent anastomosis failure. Consequently, an “open left atrium” model was adopted, in which the specimen’s pulmonary vein was left open to drain. The perfusate was allowed to drain and was subsequently collected in an XVIVO Organ Chamber^®^ (Xvivo Perfusion, Mölndal, Västra Götaland County, Sweden).

### Setup of Ex Vivo Lung Perfusion (EVLP)

**Fig. 2:**
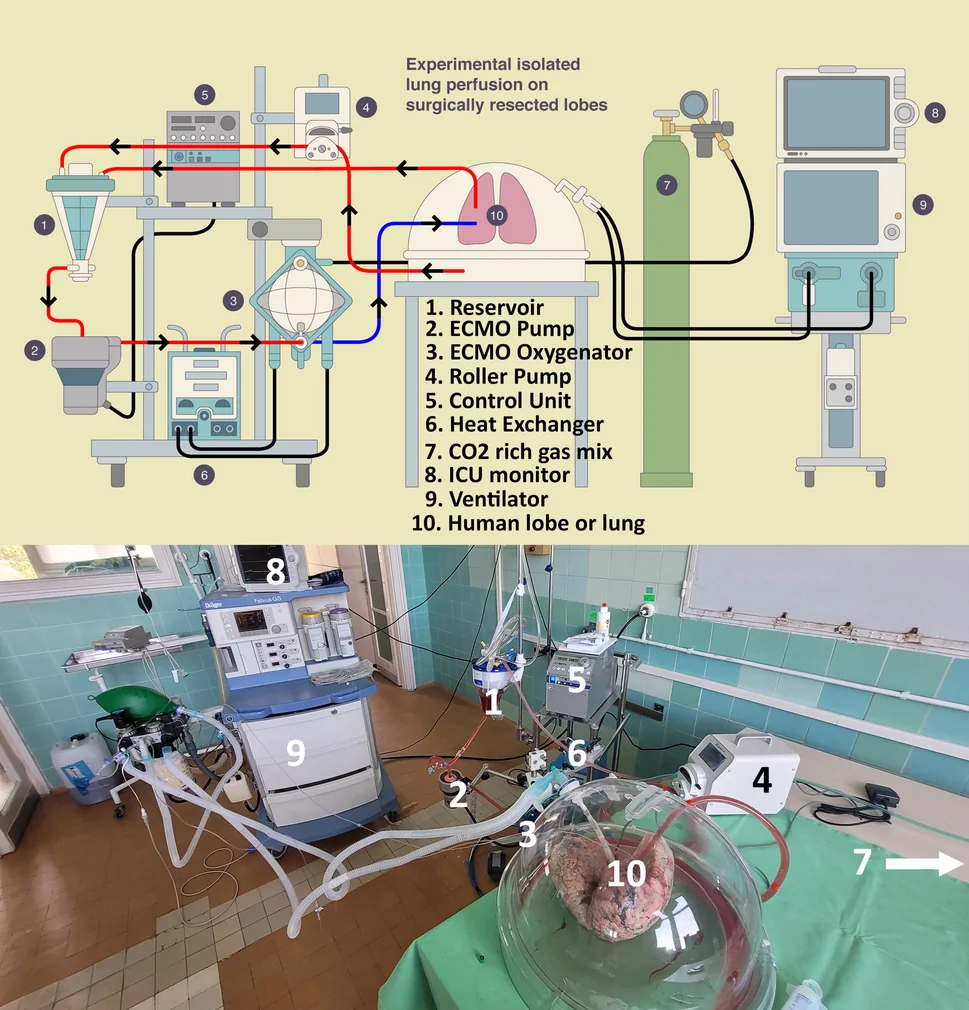
Schematic representation and practical implementation of the ex vivo lung perfusion platform used in our experiments. Red lines indicate perfusate with a high O2 concentration, while blue lines indicate perfusate with a high CO2 concentration. Various gases flow through the black lines

Our circuit was created using a Maquet Rotaflow^®^ (Getinge, Göteborg, Västra Götaland County, Sweden) and Maquet PLS^®^ (Getinge, Göteborg, Västra Götaland County, Sweden) system as seen on Fig. 2. The circuit was primed with 1 L of Steen^®^ solution (Xvivo Perfusion, Mölndal, Västra Götaland County, Sweden) stored at 4 °C. Once the preserved lung was ready for connection, we placed it inside an XVIVO Organ Chamber^®^ (Xvivo Perfusion, Mölndal, Västra Götaland County, Sweden) and connected the artery cannula to the circuit shown in Fig. 2.

We adjusted the flow to the patient’s estimated cardiac output. For lower lobectomies, utilisation of 25% (approximately 1100–1300 ml/min) of the patient’s cardiac output (CO) was employed, while for pneumonectomies, the targeted utilisation was 50% (2000–2500 ml/min) of the patient’s cardiac output. The flow was kept constant during the experiment. The maximum permissible flow through the specimen was determined according to the following formula:

$$\begin{aligned}\mathrm{Flow}_{\mathrm{max}}\;(\mathrm{mL/min})\;&=\;\text{calculated CO}\;\times\;0.4\;\\&\times\;(\text{number of segments}\;/\;19)\end{aligned}$$

After connection, the lobes were gradually warmed by incrementally increasing flow over the first 20 min to the calculated maximum based on the patient’s cardiac output and the size of the resected lobe. Ventilation commenced once normothermia (35–37 °C) was achieved within the first 20 min, and the perfusate was deoxygenated. We used volume-controlled ventilation with a maximum airway pressure (P_max_) of 25 cmH₂O, a positive end-expiratory pressure (PEEP) of 5 cmH₂O, a respiratory rate of 12 breaths/min, and an inspired oxygen fraction (FiO₂) of 21%. The target ventilation volume was calculated with the following formula: ventilation target (ml) = 7 × ideal body weight × (number of segments/19).

We placed a pressure sensor catheter into the artery line of EVLP for continuous monitoring, to sustain a mean pulmonary artery pressure between 15 and 20 mmHg.

A commercially available blood gas analyser (Roche Cobas B221, Roche, Emil-Barell-Str., Baden-Württemberg, Germany) was used to measure physiological data during the experiments with FiO_2_ set at 21%. 2 ml of samples were drawn from the Steen^®^ solution and analysed before the lung was integrated into the circuit and at 15–20-minute intervals thereafter. The solution’s pH was targeted at 7.4 and maintained by adding 8.4% sodium bicarbonate puffer. The experimental endpoint was to determine the tolerance limits of the specimens and sustain perfusion for at least 120 min, given that data on this subject is scarce. The most limiting factor was oedema formation inside the specimen. The development of dense lung parenchyma and fluid in the endobronchial tube was an indicator of rapidly deteriorating lung function. When target ventilation and perfusate flow parameters -described previously- could not be kept within physiological range or when the circulating perfusate volume was depleted by absorption, we terminated the experiment.

### Software and statistics

Physiological parameters were plotted and utilised to construct linear regression models employing the method of least squares approximation, facilitated by the statistical software R (version 4.3.2). The following R packages were utilised: ggplot2, dplyr, lme4, tidyverse. To compare pneumonectomy and lobectomy parameters, a linear mixed-effects model was employed. When analysing the results, not all cases were included for each parameter. For pCO₂ and pO₂ measurements, experiment #2 and experiment #3 were excluded due to a mid-experiment replacement of the sweep gas tank, which slightly altered readings. For lactate and glucose analysis, experiment #1, experiment #8, and experiment #9 were excluded, as external glucose was administered during these experiments. A linear mixed-effects model could not be applied to the osmolality data due to the limited number of patients in the lobectomy group.

## Results

The median age and height of the patients were 68 years and 1.74 m, respectively (range: 53–74 and 1.48–1.97, respectively). There were 6 male patients and 4 female patients. In both genders, half of the cases were staged as Stage III postoperatively. Warm ischemic time begins when arterial or venous flow is interrupted (e.g., by clamping, ligature) and ends once the lung is perfused with 4 °C Perfadex^®^ solution. Cold ischemic time then commences and continues until flow is re-established within the specimen. The median warm ischemic time was 37 min (range: 10–86 min), while the median cold ischemic time was 60 min (range: 28–95 min). The experiment was characterised as starting when flow commenced within the specimen and concluding when the flow ended. In our cohort, the median duration of the experiment time was 159 min (range: 133–228 min). In two lobectomy cases, the arteries and veins were transected within five minutes of each other, whereas in the remaining two cases, the veins were stumped 44 and 48 min prior to the arterial transection. The median warm and cold ischemic times for lobectomies were 60.5 and 49.5 min, respectively. In all pneumonectomy cases, arterial and venous divisions were performed within 10 min of each other. The median warm and cold ischemic times for pneumonectomies were recorded as 29.5 and 75 min, respectively.

During the experiment, the median lactate level increased from 0.36 mmol/L to 4.29 mmol/L, while the median glucose level decreased from 5.04 mmol/L to 3.18 mmol/L. At the start and end of the experiment, the median pH of the perfusate was 7.2 and 7.31, median sodium levels were 146.15 mmol/L and 166.85 mmol/L, and median potassium levels were 3.94 mmol/L and 3.99 mmol/L.

**Fig. 3:**
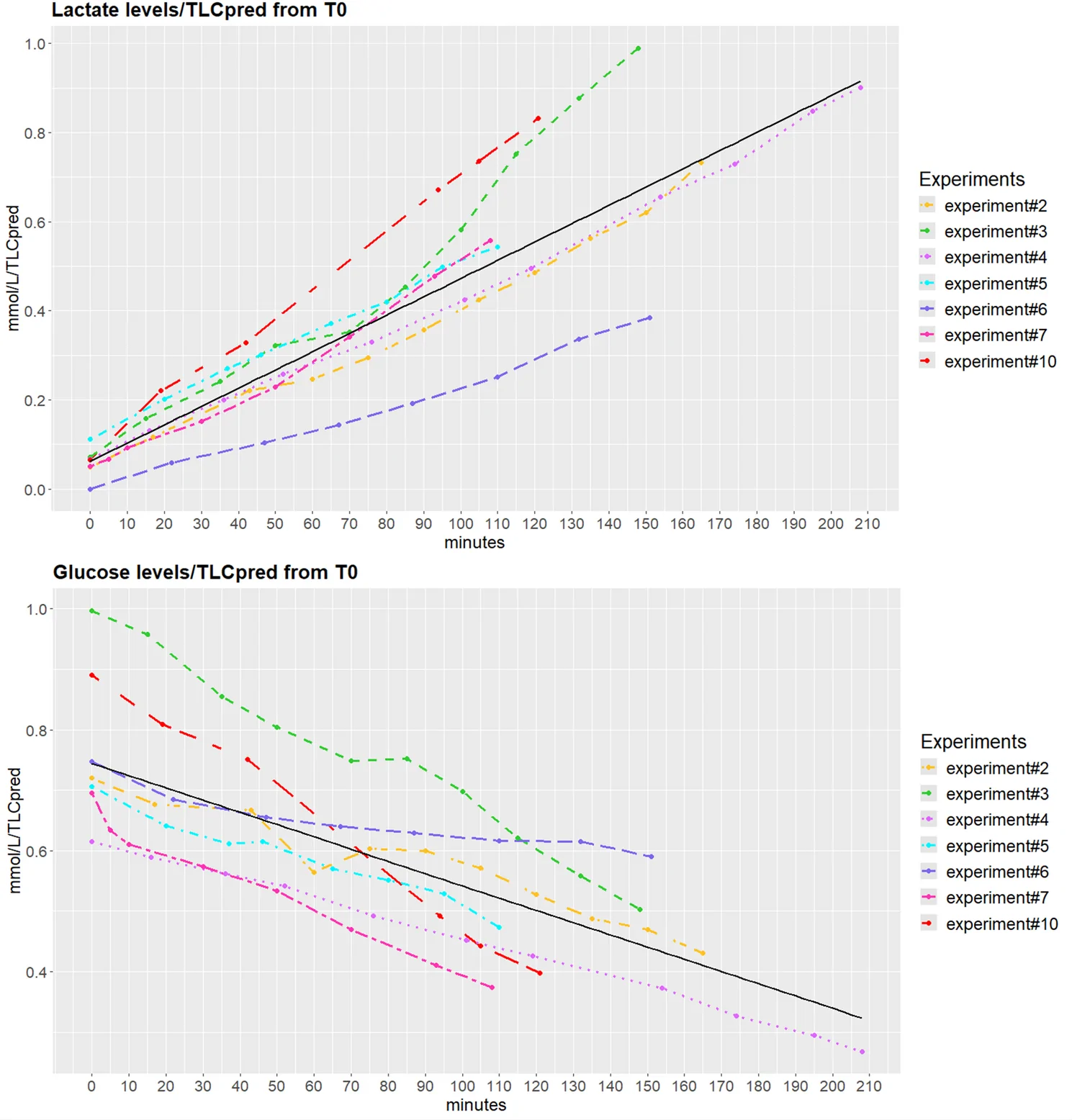
Changes in lactate and glucose levels of the perfusate during the experiment, normalized to predicted Total Lung Capacity (TLC). TLC predictions were derived from patient’s height. The black lines represent the linear regression of the experimental data. Regression formulas are respectively y = 0.0621862 + x*0.0041033 + std. error and y = 0.7442107 + x*(-0.0020247) + std. error.”

Metabolism in the specimens was characterised by lactate production and glucose consumption in the acellular perfusate. Linear regression analysis indicated significant trends for both metabolites as shown on Fig. 3. Lactate increased over time (y = 0.062 + 0.0041x; R² = 0.778, *p* < 0.001), while glucose declined (y = 0.744+(-0.0020)x; R² = 0.539, *p* < 0.001). It was observed that the absolute coefficient value for the lactate production formula closely approximated twice that of the glucose consumption formula (0.0020247 vs. 0.0041033). This alignment is congruent with the biochemical principle wherein the conversion of one glucose molecule yields the production of two lactate molecules [15]. We present the aforementioned factors on Fig. 3. Given lactate’s acidic nature, it necessitated additional pH correction measures [1, 16]. This was accomplished through incorporating a sodium bicarbonate buffer injection into our solution via ports on the tubing. During the experiments, approximately three to five ampoules of 8.4% sodium bicarbonate were used.

**Fig. 4:**
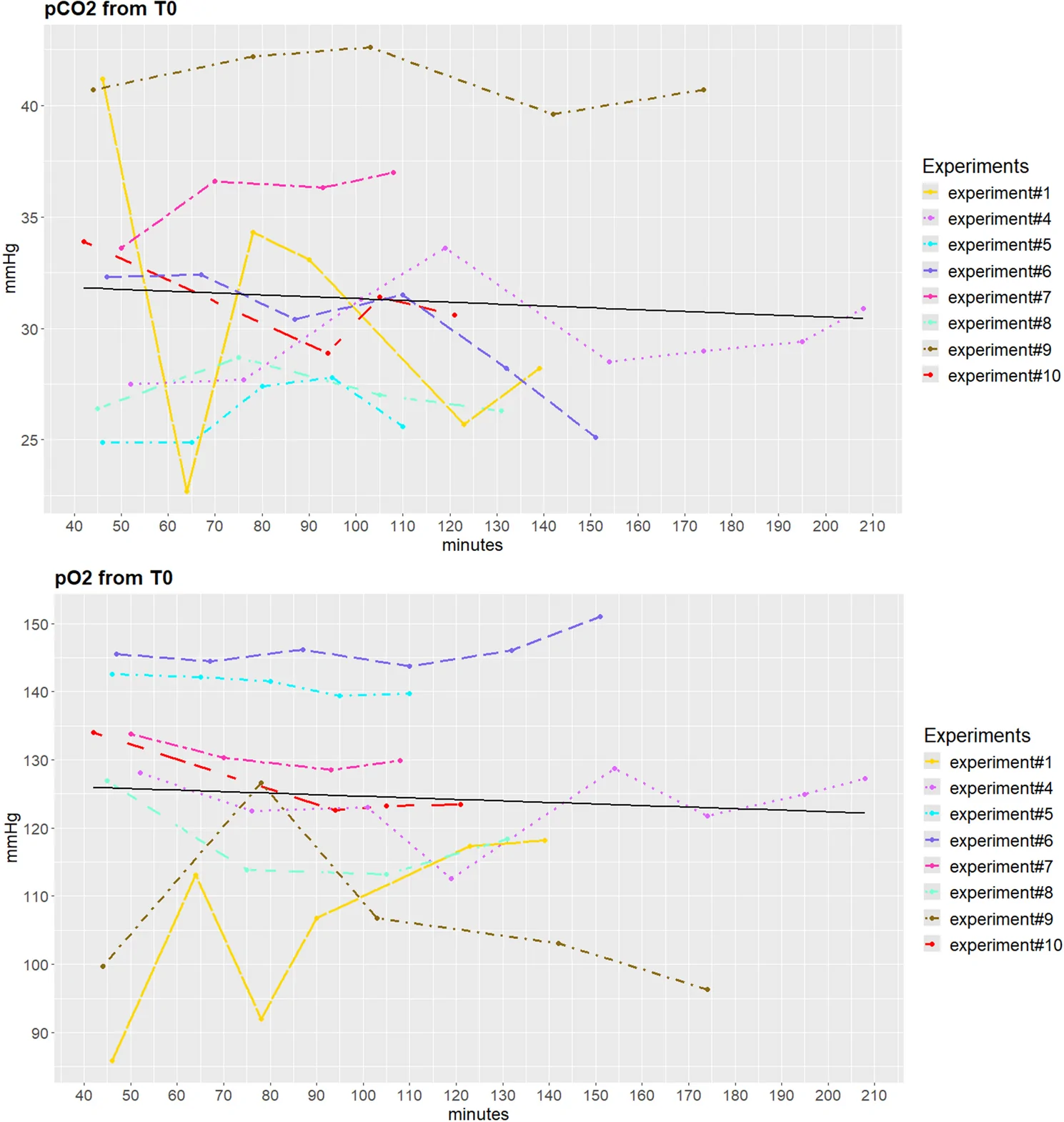
“Temporal changes in experimental lungs’ pCO2 and pO2 levels measured in the pulmonary vein. The black lines represent the linear regression of the experimental data. Regression formulas are respectively y = 32.16 + x*(-0.0083) + std. error and 126.91 + x*(-0.02265) + std. error”

The median partial pressure of carbon dioxide (pCO₂) increased from 17.35 mmHg to 29.4 mmHg, while the median partial pressure of oxygen (pO₂) decreased from 163.4 mmHg to 125.4 mmHg over the course of the experiments. The corresponding standard deviations were 7.52 mmHg for pCO₂ and 22.66 mmHg for pO₂. We present temporary changes in pO2 and PCO2 levels in Fig. 4. Our linear regression analysis of the experimental data revealed non-significant decrease in pO_2_ (y = 126.91 − 0.023x; R² = 0.004, *p* = 0.701) and in pCO_2_ (y = 32.16 − 0.008x; R² = 0.005, *p* = 0.673) levels within the pulmonary veins. However, these values remained closely aligned with human physiological conditions, indicating that gas exchange parameters remained acceptable throughout the experiment.

**Fig. 5:**
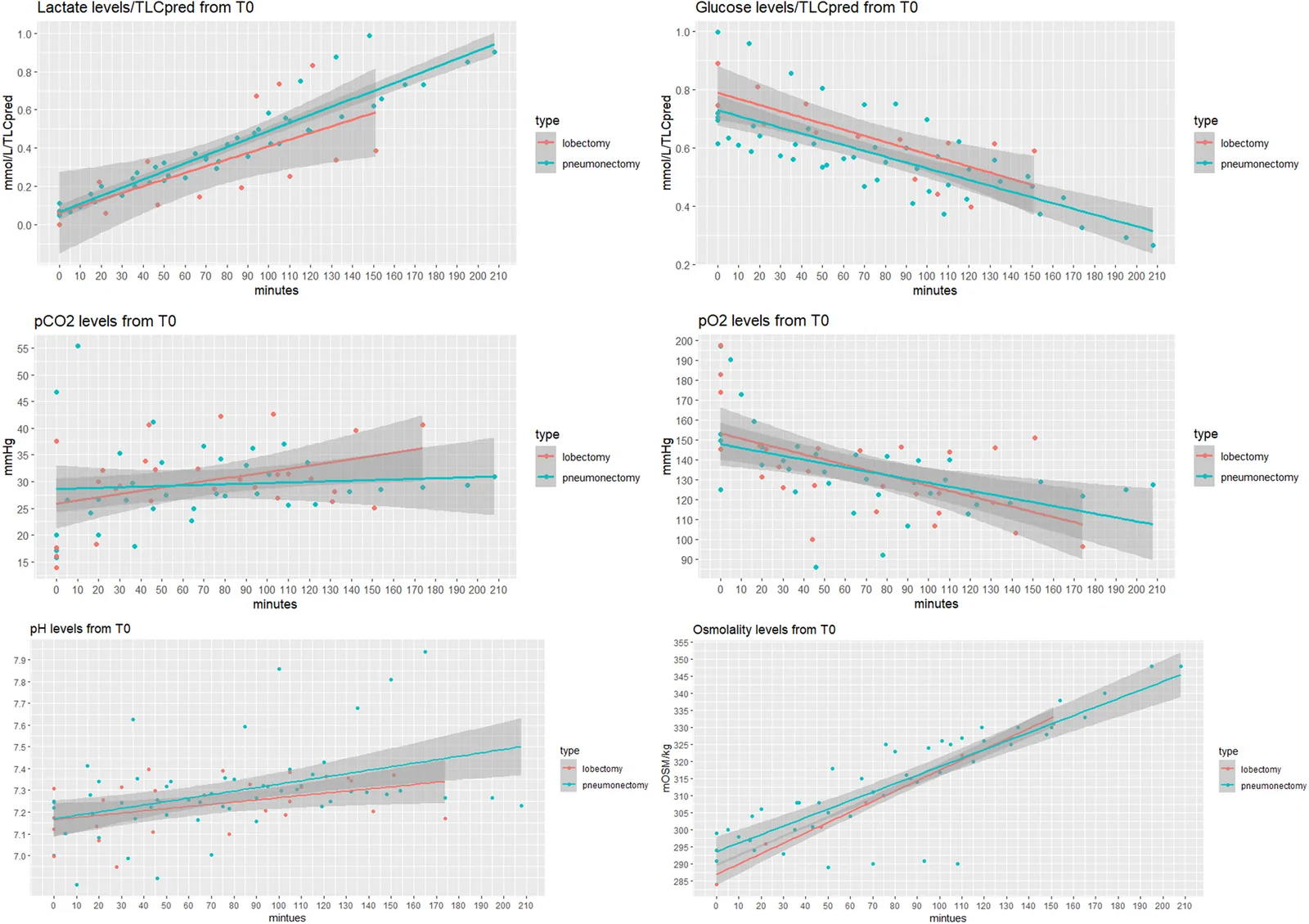
Temporal changes of physiological parameters in the ex-vivo experiments from pneumonectomy and lobectomy. The blue and red colors represent experimental data from pneumonectomy and lobectomy, respectively

There was no statistically significant temporal parameter changes observed between lobectomies and pneumonectomies using linear mixed model in relation to the following variables: glucose levels (pneumonectomy: y = 0.7298+(-0.001988*x), R²=0.535, *p* = 0.000; lobectomy: y = 0.7897+(-0.002102)*x, R²=0.6, *p* = 0.0011), (interaction p-value = 0.712); lactate levels (pneumonectomy: y = 0.0664 + 0.004221*x, R²=0.897, *p* = 0.000; lobectomy: y = 0.0614 + 0.003478*x, R²=0.436, *p* = 0.0101), (interaction p-value = 0.287); pH (pneumonectomy: y = 7.169 + 0.0016x, R²=0.182, *p* = 0.001; lobectomy: y = 7.165 + 0.0010x, R²=0.180, *p* = 0.024), (interaction p-value = 0.513); pCO_2_ levels (pneumonectomy: y = 31.1959+(-0.008692)*x, R²=0.007, *p* = 0.697; lobectomy: y = 32.977+(-0.004135)*x, R²=0.001, *p* = 0.9102), (interaction p-value = 0.416); and pO_2_ levels (pneumonectomy: y = 122.7829 + 0.011171*x, R²=0.001, *p* = 0.874, lobectomy: y = 133.2803+ (-0.079398)*x, R²=0.032, *p* = 0.4665), (interaction p-value = 0.073) as shown on Fig. 5. The median experiment length was 159 min for pneumonectomy and 162 min for lobectomy specimens. The median warm ischemic time for pneumonectomy was 29.5 min while for lobectomies it was 75 min. Comparative analyses between lobectomy and pneumonectomy specimens should be interpreted with caution, as the limited sample size prohibited robust statistical inference. The analyses are exploratory in nature.

Specimen weight was measured after Perfadex perfusion prior to the experiment and again after termination of the experiment. The cohort comprised three pneumonectomy and three lobectomy specimens. The median preoperative weights for lobectomies and pneumonectomies were 223 g and 475 g, respectively. The median postoperative weights for lobectomies and pneumonectomies were 489 g and 776 g, respectively. The median weight gain was 117 g in the pneumonectomy specimens and 266 g in the lobectomy specimens. The median relative weight gains were 2.193 and 1.246 for lobectomies and pneumonectomies respectively. Weight gain for each case is presented in Table 2.

**Table 2:** Specimens’ weight changes

Patient	Patient #3	Patient #4	Patient #5	Patient #6	Patient #8	Patient #9
Operation type	pneumonectomy	pneumonectomy	pneumonectomy	lobectomy	lobectomy	lobectomy
Weight before the experiment (gram)	475	661	451	223	312	140
Weight after the experiment (gram)	592	776	896	489	489	419
Weight gain (gram)	117	115	445	266	177	279
Relative gain	1.246	1.174	1.987	2.193	1.567	2.993

## Discussion

The application of EVLP on cancer-bearing resected lungs or lobes represents a novel approach in translational lung science and lung oncology. Initially, the method was developed to evaluate lungs from organ donors without cardiovascular function (donation after circulatory death - DCD). Due to the growing demand for organs, this technique has attracted considerable attention within the transplant field [2, 17, 18]. Several protocols were developed in side-by-side, with the Lund, Toronto, and Organ Care System protocols being the most commonly used [2]. 

We hypothesised that, beyond conventional physiological parameters reflecting lung function, such as lung compliance and pulmonary vascular resistance, malignancies may have influenced the specimens’ tolerance. The Warburg effect associated with cancer metabolism could alter lactate production in EVLP systems with cancer-bearing specimens [15]. High lactate levels were considered a potential indicator of declining organ function. Terumoto Koike et al. studied lactate and other metabolite kinetics in transplanted and rejected EVLP specimens utilizing Toronto protocol. They reported increased lactate production in both groups; however, their analysis did not account for lung size, which may significantly influence lactate levels given the fixed volume of the perfusion system. Without normalisation for the specimen’s size we are unable to directly compare with our results [19]. Alexis Slama et al. corrected for lung size and reported a comparable lactate production slope with stable pO2 and pCO2 levels, although their fluid management with Tham-Köhler buffer is superior compared to our sodium bicarbonate [1]. Our results show that, despite reaching high lactate levels, pCO2, and pO2, and ion levels remained manageable, although pCO2 and pO2 showed a slightly non-significant temporal trend.

However, we found oedema formation may be a more critical limiting factor. Fluid accumulation led to a continuous decrease in the volume of circulating solution, at times almost depleting the reservoir. In certain cases, a two- to three-fold increase in specimen weight was observed, as can be seen in Table 2. This phenomenon has been reported previously for clinical EVLP [1, 6, 11]. Weight gain data are essential for refining perfusion protocols, and to our knowledge, this study is the first to report such information in cancer-bearing human lung specimens. Compared to both animal and human EVLP models, our specimens demonstrated considerably higher weight gain signaling the need for better perfusion protocol [20, 21]. Hyperoncotic perfusates are recommended to reduce and delay oedema formation in the specimens. This observation has prompted the development of an acellular hyperoncotic perfusate: Steen Solution (Xvivo Perfusion, Mölndal, Västra Götaland County, Sweden), which is routinely used in clinical settings [2, 22]. Another potential strategy to mitigate oedema formation is the use of the Tham-Köhler buffer for pH correction as mentioned earlier. This buffer is generally preferred over sodium bicarbonate, as it effectively stabilises pH without increasing sodium levels in the perfusate. Regrettably, it was not obtainable in Hungary when the experiments were performed. The addition of sodium bicarbonate to the Steen solution had the undesirable effect of elevating both the osmolality and sodium levels of the perfusate. The slight, non-significant decrease seen in pCO_2_ and pO_2_ levels is likely also attributable to oedema formation. There remains considerable potential for improving oedema management, as other centers have maintained EVLP for up to six hours in porcine models [23]. In our preliminary cancerous EVLP studies, adopting the open left atrial model appeared beneficial for mitigating oedema formation, but it remains a controversial topic in the research community [24, 25]. 

In our cohort, five cases were ultimately diagnosed as stage III cancer following pathological examination of the resected lung and lymph nodes. These cases were initially classified at a lower stage due to undetected cancerous mediastinal lymph nodes on CT scans. Compared with the cohort published by Alxesis et al., in which only 2 of 10 cases were staged as pT3, although one case of pneumonia. It should be noted that patient recruitment was during the Coronavirus disease 2019 (COVID-19) pandemic, which limited the availability for medical screening and medical check-ups, and other resources normally allocated to pulmonologists and thoracic surgeons. Oedema formation may be influenced by the stage of the disease. In a separate experimental study, 5 specimens out of 6 from patients with suspected or confirmed Stage I/II lung cancer were successfully perfused for 140 min without significant oedema formation [6]. Determining the most appropriate tumour type and stage for experimental use remains an important consideration.

While previous human studies focused solely on utilising lobes for the experiment, we expanded our investigation to include lungs retrieved after pneumonectomy. Incorporating the entire lungs into the experiment offers two primary benefits: it expands the pool of potential experimental organs and simplifies the anastomoses involved in experimental preparation. In experimental research conducted on cancerous specimens, no available study has compared the efficacy of utilising lobes versus the lung for EVLP studies. We did not observe significant differences in the investigated physiological parameters between pneumonectomies and lobectomies. However, pneumonectomies were associated with substantially shorter warm ischemic times. The difference was due to the different surgical strategies used in the case of lobectomies, where often the pulmonary vein was cut first, essentially starting warm ischemia. In our cohort, the median warm ischemic time was comparable to other experimental settings [1, 6]. We consider a maximum of 60 min of warm ischemia to be acceptable for this type of experiment. Due to the complexity of lobectomies, particularly VATS lobectomies, we lost some cases to prolonged warm ischemic times. Tolerable cold ischemic times are significantly higher, with durations of up to 12–24 h [23, 26]. Ongoing efforts are focused on extending cold ischemic times and enhancing lung preservation techniques [26, 27]. 

For future experiments, it would be desirable to characterise the circulation within the organ. Additional research is necessary to visualise dynamic circulation parameters using CT imaging. The limited space in the CT machine makes this method difficult.

This study has several limitations. Temporal data on ventilation and perfusion volumes, airway pressures, and ventilation parameters were not collected during the experiments. The small sample size of our cohort does not allow for categorical statistical analysis, such as stage of the disease, tumour type, and tumour location. It is important to note that the scarcity of suitable cases is a major limitation, so this type of innovative perfusion system can be most efficiently conducted in thoracic surgery centers. Further metanalysis on human EVLP system with identical protocols could elucidate important factors such acceptable level of edema formation. Unfortunately, we did not measure wet-to-dry mass ratio of the collected tissues.

## Conclusion

We have demonstrated the technical feasibility of performing EVLP in resected human lung cancer specimens. It marks a preliminary step toward EVLP adaptation in Hungary, and a broader understanding of translational lung research. However, additional optimisation and validation will be necessary before EVLP on cancer-bearing specimens can be considered a robust platform for translational lung cancer research techniques. During our experiments, oedema formation posed a significant challenge, highlighting the need for improved fluid management and better experimental protocols. Maintaining near-physiological conditions in our cancer-bearing EVLP model is challenging, and the duration for which this can be sustained remains limited. The ideal patient selection criteria will be needed for further standardisation in future investigations. In our cohort, pneumonectomies and lobectomies yielded comparable physiological outcomes. The surgical complexity associated with lobectomies sometimes increases the risk of warm ischemia. Additional research utilising advanced imaging modalities will be crucial for refining EVLP protocols. The present study primarily establishes the technical feasibility of this approach; additional optimisation and validation will be necessary before it can be considered a robust platform for translational lung cancer research and pharmaceutical development.

## Data Availability

The data and materials that support the findings of this study are available on request from the corresponding author upon reasonable request.

## Abbreviations

EVLP: Ex Vivo Lung Perfusion
PET-CT: Positron emission tomography-computed tomography
FDG: Fluorodeoxyglucose
CT: Computed tomography
VATS: Video-assisted thoracoscopic surgery
S6: Apical segment of the lower lobe
CO: Cardiac output
P_max_: Maximum airway pressure
Flow_max_: Maximum permissible flow through the specimen
PEEP: Positive end-expiratory pressure
FiO₂: Inspired oxygen fraction
pCO₂: Partial pressure of carbon dioxide
pO₂: Partial pressure of oxygen
DCD: Donation after circulatory death
COVID-19: Coronavirus disease 2019
